# {μ-2-[(Benzothia­zol-2-yl-2κ*N*)hydrazonomethyl-2κ*N*]-6-methoxy­phenolato-1:2κ^3^ *O* ^1^,*O* ^6^:*O* ^1^}{2-[(benzo­thia­zol-2-yl-1κ*N*)hydrazonomethyl-1κ*N*]-6-methoxy­phenolato-1κ*O* ^1^}(methanol-2κ*O*)(nitrato-2κ*O*)dicopper(II) nitrate

**DOI:** 10.1107/S1600536809031705

**Published:** 2009-08-15

**Authors:** Yu-Ching Lin, Fung-E Hong

**Affiliations:** a Department of Chemistry, National Chung Hsing University, Taichung 402, Taiwan

## Abstract

The title complex, [Cu_2_(C_15_H_12_N_3_O_2_S)_2_(NO_3_)(CH_3_OH)]NO_3_, has two Cu^II^ centres coordinated by two deprotonated 2-[(benzothia­zol-2-yl)hydrazonometh­yl]-6-methoxy­phenol ligands, a methanol mol­ecule and a nitrate ion. Both Cu^II^ centres are penta­coordinated in a distorted square-pyramidal fashion. The crystal structure is stabilized by N—H⋯O and O—H⋯O hydrogen bonds.

## Related literature

For the preparation of the ligand, see: Patil *et al.* (2009 ▶).
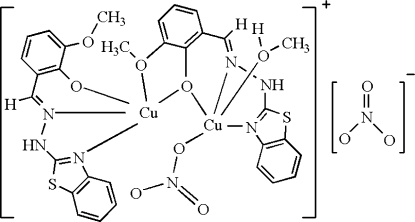


## Experimental

### 

#### Crystal data

[Cu_2_(C_15_H_12_N_3_O_2_S)_2_(NO_3_)(CH_4_O)]NO_3_
*M* *_r_* = 879.81
Monoclinic, 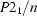

*a* = 11.6893 (12) Å
*b* = 18.9172 (18) Å
*c* = 16.8910 (17) Å
β = 91.869 (2)°
*V* = 3733.1 (6) Å^3^
*Z* = 4
Mo *K*α radiation
μ = 1.32 mm^−1^
*T* = 298 K
0.60 × 0.50 × 0.20 mm

#### Data collection

Oxford KM-4-CCD/Sapphire diffractometer
Absorption correction: multi-scan (*SADABS*; Sheldrick, 1996 ▶) *T*_min_ = 0.505, *T*_max_ = 0.778
16985 measured reflections
7175 independent reflections
5506 reflections with *I* > 2σ(*I*)
*R*_int_ = 0.028

#### Refinement

*R*[*F* ^2^ > 2σ(*F* ^2^)] = 0.044
*wR*(*F* ^2^) = 0.131
*S* = 1.01
7175 reflections
491 parameters
H atoms treated by a mixture of independent and constrained refinement
Δρ_max_ = 0.73 e Å^−3^
Δρ_min_ = −0.76 e Å^−3^

### 

Data collection: *SMART* (Bruker, 1999 ▶); cell refinement: *SAINT* (Bruker, 1999 ▶); data reduction: *SAINT*; program(s) used to solve structure: *SHELXS97* (Sheldrick, 2008 ▶); program(s) used to refine structure: *SHELXL97* (Sheldrick, 2008 ▶); molecular graphics: *SHELXTL* (Sheldrick, 2008 ▶); software used to prepare material for publication: *SHELXTL*.

## Supplementary Material

Crystal structure: contains datablocks I, global. DOI: 10.1107/S1600536809031705/bt5014sup1.cif

Structure factors: contains datablocks I. DOI: 10.1107/S1600536809031705/bt5014Isup2.hkl

Additional supplementary materials:  crystallographic information; 3D view; checkCIF report

## Figures and Tables

**Table 1:** Hydrogen-bond geometry (Å, °)

*D*—H⋯*A*	*D*—H	H⋯*A*	*D*⋯*A*	*D*—H⋯*A*
N2—H2*A*⋯O11	0.86	1.92	2.730 (4)	156
N5—H5*A*⋯O9^i^	0.86	1.90	2.728 (4)	160
O12—H12*B*⋯O1	0.77 (6)	2.04 (5)	2.763 (4)	157 (5)
O12—H12*B*⋯O2	0.77 (6)	2.44 (5)	3.025 (4)	134 (5)

Symmetry code: (i) 
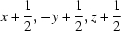
.

**Fig. 1.**
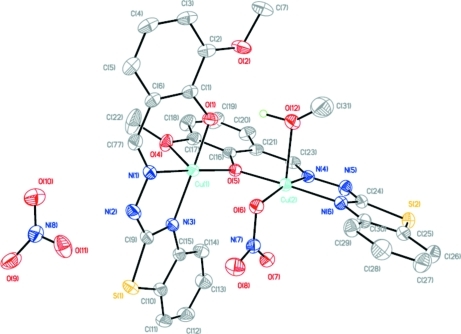
A view of the molecular structure of the title compound with displacement ellipsoids shown at the 20% probability level. Hydrogen atoms have been omitted for clarity.

## supplementary crystallographic information

### Comment

Dinuclear copper complexes chelated by ligands with biological activity are of interest to many because of their relevance to the active sites of some characterized metalloenzymes. We report herein the synthesis and crystal structure of a bis-*N*,*N*,*O*-tridentate ligand chelated dinuclear copper complex, a potential model for biologically relevant studies.

The structure of the title compound reveals that it is a bis-*N*,*N*,*O*-ligands chelated di-copper complex (Figure 1). These two copper atoms are held together by a bridging oxygen, which is deprotonated from the hydroxyl group of (2-benzothiazol-2-yl-hydrazonomethyl)-6-methoxy-phenol (Patil *et al.*, 2009). Both copper centers are penta-coordinated and their oxidation numbers are +2. The Cu(2) is coordinated by a MeOH and NO_3_^-^ besides the deprotonated ligand. The methoxyl group of the deprotonated ligand acts as the coordinating site towards Cu(1). Another deprotonated ligand is solely chelated towards Cu(1).

### Experimental

A 100 ml round-bottomed Schlenk flask equipped with a magnetic stirbar and a rubber septum was charged with a N,*N*,*O*-tridentate ligand 2-(benzothiazol-2-yl-hydrazonomethyl)-6-methoxy-phenol (1) (0.30 mmol, 89.8 mg) with one molar equivalent of Cu(NO_3_)_2_*^.^*3H_2_O in MeOH. After stirred at room temperature for 2 h, the solvent was removed under reduced pressure. The dark-green residue was subjected to various spectroscopic methods as well as to grow crystals in MeOH. It was characterized later as the title compound (2). LRMS: m/s = 722 [M—CH_3_OH—NO_3_^-^]^+^; Anal. Calcd.: S, 7.85; N, 12.00; C, 45.58; H, 7.85; Found: S, 7.19; N, 11.94; C, 41.54; H, 7.19.

### Refinement

All   H atoms bonded to N or C were placed in geometrically idealized positions and constrained to ride on their parent atoms with N—H = 0.86Å and C—H distances in the range 0.93–0.96 Å and *U*_iso_(H)=1.2*U*_eq_(C,N). The H atom bonded to O was freely refined.

### Crystal data

**Table:**

[Cu_2_(C_15_H_12_N_3_O_2_S)_2_(NO_3_)(CH_4_O)]NO_3_	*F*(000) = 1792
*M**_r_* = 879.81	*D*_x_ = 1.565 Mg m^−^^3^
Monoclinic, *P*2_1_/*n*	Mo *K*α radiation, λ = 0.71073 Å
Hall symbol: -P 2yn	Cell parameters from 7572 reflections
*a* = 11.6893 (12) Å	θ = 2.4–26.1°
*b* = 18.9172 (18) Å	µ = 1.32 mm^−^^1^
*c* = 16.8910 (17) Å	*T* = 298 K
β = 91.869 (2)°	Parallelepiped, green
*V* = 3733.1 (6) Å^3^	0.60 × 0.50 × 0.20 mm
*Z* = 4	

### Data collection

**Table:**

KM-4-CCD/Sapphire [PLEASE CHECK; DEVICE COMPATIBLE WITH BRUKER SOFTWARE?]diffractometer	7175 independent reflections
Radiation source: fine-focus sealed tube	5506 reflections with *I* > 2σ(*I*)
graphite	*R*_int_ = 0.028
φ and ω scans	θ_max_ = 26.1°, θ_min_ = 2.1°
Absorption correction: multi-scan (*SADABS*; Sheldrick, 1996)	*h* = −14→8
*T*_min_ = 0.505, *T*_max_ = 0.778	*k* = −19→22
16985 measured reflections	*l* = −20→20

### Refinement

**Table:**

Refinement on *F*^2^	Primary atom site location: structure-invariant direct methods
Least-squares matrix: full	Secondary atom site location: difference Fourier map
*R*[*F*^2^ > 2σ(*F*^2^)] = 0.044	Hydrogen site location: inferred from neighbouring sites
*wR*(*F*^2^) = 0.131	H atoms treated by a mixture of independent and constrained refinement
*S* = 1.01	*w* = 1/[σ^2^(*F*_o_^2^) + (0.09*P*)^2^] where *P* = (*F*_o_^2^ + 2*F*_c_^2^)/3
7175 reflections	(Δ/σ)_max_ = 0.001
491 parameters	Δρ_max_ = 0.73 e Å^−^^3^
0 restraints	Δρ_min_ = −0.76 e Å^−^^3^

### Special details

**Table:**

Geometry. All e.s.d.'s (except the e.s.d. in the dihedral angle between two l.s. planes) are estimated using the full covariance matrix. The cell e.s.d.'s are taken into account individually in the estimation of e.s.d.'s in distances, angles and torsion angles; correlations between e.s.d.'s in cell parameters are only used when they are defined by crystal symmetry. An approximate (isotropic) treatment of cell e.s.d.'s is used for estimating e.s.d.'s involving l.s. planes.
Refinement. Refinement of *F*^2^ against ALL reflections. The weighted *R*-factor *wR* and goodness of fit *S* are based on *F*^2^, conventional *R*-factors *R* are based on *F*, with *F* set to zero for negative *F*^2^. The threshold expression of *F*^2^ > σ(*F*^2^) is used only for calculating *R*-factors(gt) *etc*. and is not relevant to the choice of reflections for refinement. *R*-factors based on *F*^2^ are statistically about twice as large as those based on *F*, and *R*- factors based on ALL data will be even larger.

### Fractional atomic coordinates and isotropic or equivalent isotropic displacement parameters (Å^2^)

**Table:**

	*x*	*y*	*z*	*U*_iso_*/*U*_eq_	
Cu2	0.16897 (3)	0.19196 (2)	0.98737 (2)	0.04552 (13)	
Cu1	0.17847 (3)	0.33814 (2)	0.88954 (2)	0.04699 (13)	
S2	0.09665 (9)	−0.01021 (5)	1.10524 (6)	0.0695 (3)	
S1	−0.03325 (9)	0.32071 (6)	0.67004 (6)	0.0722 (3)	
O5	0.26399 (15)	0.25207 (11)	0.92201 (12)	0.0458 (5)	
N3	0.1212 (2)	0.30451 (15)	0.78293 (14)	0.0502 (6)	
O6	0.03427 (17)	0.24777 (12)	0.94855 (13)	0.0524 (5)	
C6	0.1412 (3)	0.49632 (18)	0.9682 (2)	0.0560 (8)	
N1	0.0858 (2)	0.42009 (15)	0.85961 (15)	0.0527 (6)	
C16	0.3787 (2)	0.25018 (17)	0.91594 (17)	0.0473 (7)	
N6	0.0880 (2)	0.11744 (14)	1.04800 (15)	0.0522 (6)	
O1	0.2106 (2)	0.37760 (12)	0.99117 (13)	0.0625 (6)	
C9	0.0410 (3)	0.34724 (18)	0.75554 (19)	0.0536 (8)	
N2	0.0180 (3)	0.40770 (16)	0.79262 (17)	0.0645 (8)	
H2A	−0.0346	0.4365	0.7762	0.077*	
O8	−0.1039 (2)	0.23804 (17)	0.86045 (18)	0.0861 (9)	
N7	−0.0182 (2)	0.21226 (16)	0.89173 (16)	0.0554 (7)	
N5	0.2648 (2)	0.06424 (16)	1.03947 (17)	0.0622 (7)	
H5A	0.3114	0.0297	1.0479	0.075*	
O7	0.0236 (2)	0.15521 (14)	0.87204 (15)	0.0659 (6)	
C23	0.4013 (3)	0.13224 (18)	0.98134 (19)	0.0533 (8)	
H23A	0.4512	0.0951	0.9928	0.064*	
C24	0.1555 (3)	0.06304 (18)	1.06209 (18)	0.0546 (8)	
C21	0.4457 (2)	0.19332 (18)	0.94236 (19)	0.0523 (8)	
O2	0.3007 (2)	0.40931 (16)	1.12742 (15)	0.0803 (8)	
C77	0.0805 (3)	0.48056 (19)	0.8948 (2)	0.0573 (8)	
H7A	0.0352	0.5157	0.8715	0.069*	
O4	0.36346 (19)	0.36025 (15)	0.85199 (17)	0.0738 (7)	
C15	0.1322 (3)	0.24591 (19)	0.73275 (17)	0.0532 (8)	
C30	−0.0217 (3)	0.1033 (2)	1.07330 (19)	0.0578 (8)	
C4	0.1840 (3)	0.5837 (2)	1.0687 (3)	0.0768 (11)	
H4A	0.1802	0.6300	1.0871	0.092*	
C14	0.2113 (3)	0.1919 (2)	0.7406 (2)	0.0673 (9)	
H14A	0.2644	0.1916	0.7828	0.081*	
C1	0.1997 (3)	0.44431 (19)	1.0125 (2)	0.0563 (8)	
C2	0.2487 (3)	0.4635 (2)	1.0880 (2)	0.0632 (9)	
C20	0.5644 (3)	0.1947 (2)	0.9317 (3)	0.0716 (11)	
H20A	0.6088	0.1565	0.9489	0.086*	
C26	−0.1375 (4)	0.0105 (3)	1.1318 (2)	0.0813 (12)	
H26A	−0.1448	−0.0349	1.1523	0.098*	
C5	0.1351 (3)	0.5667 (2)	0.9977 (3)	0.0702 (10)	
H5B	0.0970	0.6012	0.9678	0.084*	
C10	0.0540 (3)	0.2456 (2)	0.66823 (19)	0.0617 (9)	
C17	0.4335 (3)	0.3070 (2)	0.8788 (2)	0.0608 (9)	
C25	−0.0335 (3)	0.0349 (2)	1.10562 (19)	0.0633 (9)	
C3	0.2409 (3)	0.5318 (2)	1.1151 (2)	0.0734 (11)	
H3A	0.2732	0.5439	1.1643	0.088*	
C18	0.5501 (3)	0.3068 (3)	0.8693 (3)	0.0833 (12)	
H18A	0.5849	0.3445	0.8443	0.100*	
C29	−0.1147 (3)	0.1487 (2)	1.0691 (2)	0.0710 (10)	
H29A	−0.1080	0.1942	1.0490	0.085*	
C28	−0.2188 (4)	0.1238 (3)	1.0962 (3)	0.0901 (13)	
H28A	−0.2825	0.1533	1.0940	0.108*	
C19	0.6154 (3)	0.2502 (3)	0.8970 (3)	0.0924 (14)	
H19A	0.6944	0.2505	0.8917	0.111*	
C22	0.4085 (4)	0.4258 (3)	0.8341 (4)	0.139 (3)	
H22A	0.4871	0.4205	0.8203	0.209*	
H22B	0.4040	0.4563	0.8793	0.209*	
H22C	0.3657	0.4460	0.7902	0.209*	
C27	−0.2291 (4)	0.0558 (3)	1.1265 (3)	0.0957 (15)	
H27A	−0.2999	0.0406	1.1435	0.115*	
N4	0.2957 (2)	0.12581 (14)	1.00154 (14)	0.0490 (6)	
O11	−0.1204 (2)	0.49196 (19)	0.69980 (17)	0.0884 (9)	
N8	−0.0457 (3)	0.52181 (17)	0.6607 (2)	0.0697 (8)	
O10	0.0387 (3)	0.5485 (2)	0.6927 (2)	0.1137 (12)	
O9	−0.0595 (3)	0.52210 (19)	0.58779 (18)	0.1112 (13)	
C13	0.2104 (4)	0.1384 (2)	0.6849 (2)	0.0814 (12)	
H13A	0.2628	0.1015	0.6901	0.098*	
C11	0.0523 (4)	0.1918 (2)	0.6117 (2)	0.0756 (11)	
H11A	−0.0007	0.1917	0.5695	0.091*	
C7	0.3442 (4)	0.4214 (3)	1.2059 (2)	0.0878 (14)	
H7B	0.3784	0.3787	1.2264	0.132*	
H7C	0.2827	0.4353	1.2389	0.132*	
H7D	0.4007	0.4582	1.2054	0.132*	
O12	0.2106 (3)	0.26246 (18)	1.09179 (16)	0.0770 (8)	
C12	0.1321 (4)	0.1391 (3)	0.6212 (2)	0.0864 (13)	
H12A	0.1339	0.1029	0.5840	0.104*	
C31	0.1538 (6)	0.2642 (4)	1.1638 (3)	0.142 (3)	
H31A	0.1884	0.2993	1.1979	0.213*	
H31B	0.1594	0.2187	1.1888	0.213*	
H31C	0.0746	0.2757	1.1538	0.213*	
H12B	0.217 (4)	0.300 (3)	1.075 (3)	0.085 (17)*	

### Atomic displacement parameters (Å^2^)

**Table:**

	*U*^11^	*U*^22^	*U*^33^	*U*^12^	*U*^13^	*U*^23^
Cu2	0.03765 (19)	0.0485 (2)	0.0502 (2)	0.00094 (14)	−0.00239 (15)	0.00548 (16)
Cu1	0.0459 (2)	0.0472 (2)	0.0473 (2)	−0.00129 (15)	−0.00812 (15)	0.00114 (16)
S2	0.0852 (6)	0.0598 (6)	0.0633 (5)	−0.0080 (5)	−0.0023 (5)	0.0163 (4)
S1	0.0756 (6)	0.0823 (7)	0.0568 (5)	−0.0011 (5)	−0.0248 (5)	0.0035 (4)
O5	0.0333 (9)	0.0516 (12)	0.0522 (11)	−0.0009 (8)	−0.0043 (8)	0.0040 (9)
N3	0.0497 (14)	0.0577 (17)	0.0431 (13)	−0.0041 (12)	−0.0030 (11)	0.0043 (12)
O6	0.0416 (10)	0.0542 (13)	0.0613 (12)	0.0022 (9)	−0.0004 (9)	0.0037 (10)
C6	0.0513 (17)	0.050 (2)	0.0670 (19)	−0.0092 (14)	0.0074 (15)	−0.0052 (16)
N1	0.0525 (14)	0.0544 (17)	0.0506 (14)	−0.0008 (12)	−0.0082 (11)	0.0068 (12)
C16	0.0336 (13)	0.059 (2)	0.0488 (16)	−0.0051 (13)	−0.0022 (12)	−0.0062 (14)
N6	0.0523 (14)	0.0516 (16)	0.0523 (14)	−0.0021 (12)	−0.0026 (11)	0.0039 (12)
O1	0.0790 (15)	0.0510 (15)	0.0562 (12)	0.0005 (12)	−0.0189 (11)	−0.0077 (11)
C9	0.0526 (17)	0.059 (2)	0.0485 (16)	−0.0012 (15)	−0.0075 (14)	0.0087 (15)
N2	0.0694 (18)	0.064 (2)	0.0592 (16)	0.0080 (15)	−0.0179 (14)	0.0060 (14)
O8	0.0420 (12)	0.109 (2)	0.106 (2)	0.0093 (14)	−0.0193 (13)	0.0145 (18)
N7	0.0357 (12)	0.0636 (19)	0.0665 (16)	−0.0058 (12)	−0.0028 (12)	0.0108 (14)
N5	0.0587 (16)	0.0533 (17)	0.0744 (18)	0.0074 (13)	−0.0016 (14)	0.0136 (14)
O7	0.0718 (15)	0.0569 (16)	0.0686 (15)	−0.0064 (12)	−0.0034 (12)	0.0010 (12)
C23	0.0426 (15)	0.056 (2)	0.0607 (18)	0.0106 (14)	−0.0063 (13)	−0.0105 (15)
C24	0.0632 (19)	0.052 (2)	0.0483 (16)	−0.0020 (15)	−0.0053 (14)	0.0049 (14)
C21	0.0378 (14)	0.059 (2)	0.0601 (18)	0.0012 (13)	−0.0024 (13)	−0.0113 (15)
O2	0.0884 (18)	0.088 (2)	0.0625 (14)	0.0065 (15)	−0.0262 (13)	−0.0214 (14)
C77	0.0533 (18)	0.050 (2)	0.069 (2)	0.0012 (14)	0.0025 (15)	0.0059 (16)
O4	0.0513 (13)	0.0722 (17)	0.0977 (19)	−0.0148 (12)	−0.0011 (13)	0.0295 (15)
C15	0.0566 (18)	0.060 (2)	0.0428 (15)	−0.0086 (15)	0.0028 (13)	0.0015 (14)
C30	0.0557 (18)	0.066 (2)	0.0514 (17)	−0.0089 (16)	0.0030 (14)	0.0011 (16)
C4	0.070 (2)	0.060 (2)	0.101 (3)	−0.0203 (19)	0.011 (2)	−0.025 (2)
C14	0.063 (2)	0.077 (3)	0.063 (2)	0.0043 (18)	0.0019 (17)	−0.0098 (18)
C1	0.0503 (17)	0.057 (2)	0.0614 (18)	−0.0097 (15)	0.0020 (14)	−0.0087 (16)
C2	0.0546 (18)	0.065 (2)	0.070 (2)	−0.0084 (16)	−0.0035 (16)	−0.0174 (18)
C20	0.0400 (16)	0.074 (3)	0.101 (3)	0.0057 (17)	−0.0024 (18)	−0.012 (2)
C26	0.083 (3)	0.087 (3)	0.075 (2)	−0.024 (2)	0.014 (2)	0.007 (2)
C5	0.067 (2)	0.051 (2)	0.094 (3)	−0.0070 (17)	0.013 (2)	−0.0040 (19)
C10	0.065 (2)	0.072 (2)	0.0476 (17)	−0.0147 (17)	−0.0009 (15)	0.0049 (16)
C17	0.0432 (16)	0.070 (2)	0.069 (2)	−0.0122 (16)	0.0021 (15)	−0.0014 (18)
C25	0.071 (2)	0.070 (2)	0.0492 (17)	−0.0137 (18)	0.0005 (15)	0.0024 (16)
C3	0.062 (2)	0.076 (3)	0.082 (2)	−0.0196 (19)	0.0017 (19)	−0.028 (2)
C18	0.0476 (19)	0.089 (3)	0.114 (3)	−0.018 (2)	0.012 (2)	0.007 (3)
C29	0.062 (2)	0.080 (3)	0.071 (2)	−0.0003 (19)	0.0125 (18)	0.007 (2)
C28	0.060 (2)	0.117 (4)	0.093 (3)	0.004 (2)	0.015 (2)	0.000 (3)
C19	0.0401 (18)	0.098 (3)	0.140 (4)	−0.007 (2)	0.010 (2)	−0.005 (3)
C22	0.083 (3)	0.106 (4)	0.229 (7)	−0.020 (3)	0.000 (4)	0.082 (5)
C27	0.079 (3)	0.119 (4)	0.091 (3)	−0.035 (3)	0.022 (2)	0.005 (3)
N4	0.0501 (13)	0.0469 (15)	0.0496 (13)	0.0015 (11)	−0.0057 (11)	−0.0001 (11)
O11	0.0606 (15)	0.123 (3)	0.0823 (17)	0.0176 (16)	0.0054 (14)	0.0302 (18)
N8	0.077 (2)	0.0537 (19)	0.078 (2)	−0.0003 (15)	−0.0022 (18)	0.0040 (16)
O10	0.113 (3)	0.099 (3)	0.127 (3)	−0.026 (2)	−0.033 (2)	−0.002 (2)
O9	0.159 (3)	0.103 (3)	0.0727 (18)	−0.077 (2)	0.011 (2)	−0.0064 (17)
C13	0.092 (3)	0.078 (3)	0.075 (2)	0.009 (2)	0.006 (2)	−0.019 (2)
C11	0.086 (3)	0.087 (3)	0.053 (2)	−0.016 (2)	−0.0083 (19)	−0.0095 (19)
C7	0.079 (3)	0.122 (4)	0.062 (2)	0.002 (3)	−0.020 (2)	−0.024 (2)
O12	0.109 (2)	0.068 (2)	0.0537 (14)	−0.0073 (17)	−0.0022 (14)	−0.0026 (14)
C12	0.105 (3)	0.085 (3)	0.070 (2)	−0.014 (3)	0.005 (2)	−0.024 (2)
C31	0.186 (7)	0.147 (6)	0.095 (4)	−0.047 (5)	0.047 (4)	−0.037 (4)

### Geometric parameters (Å, °)

**Table:**

Cu2—N4	1.948 (3)	C30—C29	1.386 (5)
Cu2—O5	1.956 (2)	C30—C25	1.413 (5)
Cu2—O6	1.989 (2)	C4—C5	1.351 (6)
Cu2—N6	1.999 (3)	C4—C3	1.409 (6)
Cu2—O12	2.252 (3)	C4—H4A	0.9300
Cu1—O1	1.898 (2)	C14—C13	1.383 (5)
Cu1—N1	1.948 (3)	C14—H14A	0.9300
Cu1—O5	1.979 (2)	C1—C2	1.428 (5)
Cu1—N3	2.004 (2)	C2—C3	1.376 (5)
Cu1—O4	2.311 (2)	C20—C19	1.352 (6)
S2—C24	1.720 (3)	C20—H20A	0.9300
S2—C25	1.745 (4)	C26—C27	1.372 (7)
S1—C9	1.734 (3)	C26—C25	1.386 (5)
S1—C10	1.750 (4)	C26—H26A	0.9300
O5—C16	1.349 (3)	C5—H5B	0.9300
N3—C9	1.311 (4)	C10—C11	1.394 (5)
N3—C15	1.404 (4)	C17—C18	1.378 (5)
O6—N7	1.308 (4)	C3—H3A	0.9300
C6—C1	1.401 (5)	C18—C19	1.387 (7)
C6—C5	1.424 (5)	C18—H18A	0.9300
C6—C77	1.440 (5)	C29—C28	1.396 (6)
N1—C77	1.291 (4)	C29—H29A	0.9300
N1—N2	1.380 (4)	C28—C27	1.392 (7)
C16—C21	1.395 (5)	C28—H28A	0.9300
C16—C17	1.409 (5)	C19—H19A	0.9300
N6—C24	1.314 (4)	C22—H22A	0.9600
N6—C30	1.390 (4)	C22—H22B	0.9600
O1—C1	1.320 (4)	C22—H22C	0.9600
C9—N2	1.336 (4)	C27—H27A	0.9300
N2—H2A	0.8600	O11—N8	1.247 (4)
O8—N7	1.219 (3)	N8—O10	1.219 (4)
N7—O7	1.235 (4)	N8—O9	1.237 (4)
N5—C24	1.345 (4)	C13—C12	1.390 (6)
N5—N4	1.383 (4)	C13—H13A	0.9300
N5—H5A	0.8600	C11—C12	1.371 (6)
C23—N4	1.298 (4)	C11—H11A	0.9300
C23—C21	1.435 (5)	C7—H7B	0.9600
C23—H23A	0.9300	C7—H7C	0.9600
C21—C20	1.405 (4)	C7—H7D	0.9600
O2—C2	1.355 (5)	O12—C31	1.405 (6)
O2—C7	1.423 (4)	O12—H12B	0.77 (5)
C77—H7A	0.9300	C12—H12A	0.9300
O4—C17	1.366 (4)	C31—H31A	0.9600
O4—C22	1.385 (5)	C31—H31B	0.9600
C15—C14	1.381 (5)	C31—H31C	0.9600
C15—C10	1.399 (4)		
			
N4—Cu2—O5	89.97 (10)	C13—C14—H14A	120.5
N4—Cu2—O6	166.43 (10)	C15—C14—H14A	120.5
O5—Cu2—O6	87.90 (8)	O1—C1—C6	125.1 (3)
N4—Cu2—N6	81.65 (11)	O1—C1—C2	116.6 (3)
O5—Cu2—N6	170.53 (10)	C6—C1—C2	118.3 (3)
O6—Cu2—N6	99.22 (10)	O2—C2—C3	125.5 (3)
N4—Cu2—O12	98.05 (11)	O2—C2—C1	114.0 (3)
O5—Cu2—O12	89.17 (11)	C3—C2—C1	120.4 (4)
O6—Cu2—O12	95.31 (11)	C19—C20—C21	121.4 (4)
N6—Cu2—O12	96.34 (12)	C19—C20—H20A	119.3
O1—Cu1—N1	90.78 (11)	C21—C20—H20A	119.3
O1—Cu1—O5	89.41 (9)	C27—C26—C25	117.5 (4)
N1—Cu1—O5	176.51 (10)	C27—C26—H26A	121.3
O1—Cu1—N3	171.04 (11)	C25—C26—H26A	121.3
N1—Cu1—N3	81.54 (11)	C4—C5—C6	120.6 (4)
O5—Cu1—N3	97.97 (10)	C4—C5—H5B	119.7
O1—Cu1—O4	91.03 (11)	C6—C5—H5B	119.7
N1—Cu1—O4	107.58 (10)	C11—C10—C15	122.0 (4)
O5—Cu1—O4	75.89 (8)	C11—C10—S1	127.5 (3)
N3—Cu1—O4	95.67 (10)	C15—C10—S1	110.4 (3)
C24—S2—C25	88.19 (17)	O4—C17—C18	123.2 (4)
C9—S1—C10	88.49 (16)	O4—C17—C16	115.8 (3)
C16—O5—Cu2	127.72 (19)	C18—C17—C16	121.0 (4)
C16—O5—Cu1	119.69 (19)	C26—C25—C30	121.7 (4)
Cu2—O5—Cu1	110.11 (9)	C26—C25—S2	127.7 (3)
C9—N3—C15	110.6 (3)	C30—C25—S2	110.5 (3)
C9—N3—Cu1	109.6 (2)	C2—C3—C4	120.3 (4)
C15—N3—Cu1	139.4 (2)	C2—C3—H3A	119.8
N7—O6—Cu2	108.46 (19)	C4—C3—H3A	119.8
C1—C6—C5	119.9 (3)	C17—C18—C19	119.9 (4)
C1—C6—C77	122.2 (3)	C17—C18—H18A	120.0
C5—C6—C77	117.8 (3)	C19—C18—H18A	120.0
C77—N1—N2	119.5 (3)	C30—C29—C28	117.7 (4)
C77—N1—Cu1	128.3 (2)	C30—C29—H29A	121.2
N2—N1—Cu1	112.2 (2)	C28—C29—H29A	121.2
O5—C16—C21	123.0 (3)	C27—C28—C29	121.3 (5)
O5—C16—C17	118.7 (3)	C27—C28—H28A	119.3
C21—C16—C17	118.2 (3)	C29—C28—H28A	119.3
C24—N6—C30	110.4 (3)	C20—C19—C18	120.0 (4)
C24—N6—Cu2	110.7 (2)	C20—C19—H19A	120.0
C30—N6—Cu2	138.6 (2)	C18—C19—H19A	120.0
C1—O1—Cu1	127.2 (2)	O4—C22—H22A	109.5
N3—C9—N2	121.0 (3)	O4—C22—H22B	109.5
N3—C9—S1	116.6 (3)	H22A—C22—H22B	109.5
N2—C9—S1	122.4 (2)	O4—C22—H22C	109.5
C9—N2—N1	114.2 (3)	H22A—C22—H22C	109.5
C9—N2—H2A	122.9	H22B—C22—H22C	109.5
N1—N2—H2A	122.9	C26—C27—C28	121.6 (4)
O8—N7—O7	124.0 (3)	C26—C27—H27A	119.2
O8—N7—O6	118.1 (3)	C28—C27—H27A	119.2
O7—N7—O6	117.8 (2)	C23—N4—N5	117.8 (3)
C24—N5—N4	114.3 (3)	C23—N4—Cu2	129.3 (2)
C24—N5—H5A	122.9	N5—N4—Cu2	112.9 (2)
N4—N5—H5A	122.9	O10—N8—O9	121.2 (4)
N4—C23—C21	123.9 (3)	O10—N8—O11	121.5 (4)
N4—C23—H23A	118.1	O9—N8—O11	117.3 (4)
C21—C23—H23A	118.1	C14—C13—C12	120.6 (4)
N6—C24—N5	120.4 (3)	C14—C13—H13A	119.7
N6—C24—S2	117.5 (3)	C12—C13—H13A	119.7
N5—C24—S2	122.1 (3)	C12—C11—C10	117.2 (4)
C16—C21—C20	119.4 (3)	C12—C11—H11A	121.4
C16—C21—C23	124.1 (3)	C10—C11—H11A	121.4
C20—C21—C23	116.5 (3)	O2—C7—H7B	109.5
C2—O2—C7	118.5 (3)	O2—C7—H7C	109.5
N1—C77—C6	123.4 (3)	H7B—C7—H7C	109.5
N1—C77—H7A	118.3	O2—C7—H7D	109.5
C6—C77—H7A	118.3	H7B—C7—H7D	109.5
C17—O4—C22	120.3 (3)	H7C—C7—H7D	109.5
C17—O4—Cu1	109.36 (19)	C31—O12—Cu2	126.5 (3)
C22—O4—Cu1	126.0 (3)	C31—O12—H12B	111 (4)
C14—C15—C10	119.4 (3)	Cu2—O12—H12B	106 (4)
C14—C15—N3	126.9 (3)	C11—C12—C13	121.8 (4)
C10—C15—N3	113.8 (3)	C11—C12—H12A	119.1
C29—C30—N6	126.5 (3)	C13—C12—H12A	119.1
C29—C30—C25	120.1 (3)	O12—C31—H31A	109.5
N6—C30—C25	113.4 (3)	O12—C31—H31B	109.5
C5—C4—C3	120.4 (4)	H31A—C31—H31B	109.5
C5—C4—H4A	119.8	O12—C31—H31C	109.5
C3—C4—H4A	119.8	H31A—C31—H31C	109.5
C13—C14—C15	119.1 (4)	H31B—C31—H31C	109.5

### Hydrogen-bond geometry (Å, °)

**Table:**

*D*—H···*A*	*D*—H	H···*A*	*D*···*A*	*D*—H···*A*
N2—H2A···O11	0.86	1.92	2.730 (4)	156
N5—H5A···O9^i^	0.86	1.90	2.728 (4)	160
O12—H12B···O1	0.77 (6)	2.04 (5)	2.763 (4)	157 (5)
O12—H12B···O2	0.77 (6)	2.44 (5)	3.025 (4)	134 (5)

Symmetry codes: (i) *x*+1/2, −*y*+1/2, *z*+1/2.
